# Relationship between glycemic control and cognitive impairment: A systematic review and meta-analysis

**DOI:** 10.3389/fnagi.2023.1126183

**Published:** 2023-01-26

**Authors:** Yufeng Lin, Zhongying Gong, Chunchao Ma, Zhiyun Wang, Kaiyuan Wang

**Affiliations:** ^1^Department of Neurology, Tianjin First Central Hospital, School of Medicine, Nankai University, Tianjin, China; ^2^Department of Anesthesiology, Tianjin Medical University Cancer Institute & Hospital, National Clinical Research Center for Cancer, Tianjin’s Clinical Research Center for Cancer, Key Laboratory of Cancer Immunology and Biotherapy, Tianjin, China

**Keywords:** diabetes mellitus, hyperglycemia, antidiabetic drugs, cognitive impairment, meta-analysis

## Abstract

**Background:**

Diabetes mellitus, or hyperglycemia, is an independent risk factor for cognitive impairment. Here we systematically analyzed whether glycemic control could improve cognitive impairment in patients with diabetes mellitus (DM), hyperglycemia, or insulin resistance.

**Methods:**

Three databases (PubMed, EMBASE, and Cochrane Library) and ClinicalTrials.gov were searched for randomized controlled trials analyzing the relationship between glycemic control and cognitive function assessments, published from database inception to June 2022. Patients in experimental groups were treated with antidiabetic drugs, while control groups were treated with a placebo or alternative antidiabetic drugs. Data analysis was conducted using RevMan 5.3 and StataSE-64, and standardized mean difference (SMD) and 95% confidence intervals (CIs) were calculated.

**Results:**

Thirteen studies comprising 19,314 participants were included. Analysis revealed that glycemic control significantly attenuated the degree of decline in cognitive function assessment scores (SMD  =  0.15; 95% CI 0.05, 0.26; *p * <  0.00001), and funnel plots confirmed no publication bias. Seven studies used Mini-Mental State Examination as the primary cognitive function assessment, showing that glycemic control significantly delayed the degree of decline in cognitive function assessment scores (SMD  =  0.18; 95% CI 0.03, 0.34; *p * =  0.02). Similar results were seen in two studies using the Montreal Cognitive Assessment scale, but without significant difference (SMD  =  0.05; 95% CI-0.10, 0.21; *p * =  0.51). One study using Auditory Word Learning Test (AVLT) showed that glycemic control significantly delayed the decline in cognitive function assessment scores (SMD  =  0.52; 95% CI 0.11,0.93; *p * =  0.01), and another used Wechsler Memory Scale Revised, showing similar results (SMD  =  1.45; 95% CI 0.86, 2.04; *p * <  0.00001). Likewise, a study that used Modified Mini-Mental State scale showed that glycemic control significantly delayed the decline in cognitive function assessment scores (SMD  =  -0.10; 95% CI-0.16, −0.03; *p * =  0.005). Lastly, one study used AVLT subtests to show that glycemic control delayed the decline in cognitive function assessment scores, although not statistically significant (SMD  =  0.09; 95% CI-0.53, 0.71; *p*  =  0.78).

**Conclusion:**

Glycemic control through antidiabetic treatment correlates with the improvement of cognitive impairment in patients with DM, hyperglycemia or insulin resistance. However, further studies are needed to validate the results of this study.

**Systematic Review Registration:**

PROSPERO, identifier CRD42022342260.

## 1. Introduction

Hyperglycemic conditions, particularly diabetes mellitus (DM), are strongly associated with the incidence of cognitive impairment, including both mild cognitive impairment and dementia (Biessels and Despa, 2018; van Sloten et al., 2020). Chronic peripheral hyperinsulinemia and insulin resistance are the main features of DM, but hyperglycemia is increasingly thought to be the cause of cognitive impairment in elderly patients with DM (Umegaki et al., 2017; Tahmi et al., 2021). Several studies have shown that patients with Alzheimer’s disease (AD) have desensitized insulin signals in their brains, even in the absence of DM (Jash et al., 2020). Extensive abnormalities in insulin and insulin-like growth factor type I and II (IGF-I and IGF-II) signaling pathways in the brains of patients with AD suggest that AD may partially share characteristics with a neuroendocrine disease similar to DM (Xu et al., 2015). Chronic peripheral hyperinsulinemia can cause brain insulin resistance and defective insulin receptor activity by impairing the blood–brain barrier and insulin transport to the brain (He et al., 2020; Milstein and Ferris, 2021). Therefore, impaired brain insulin signaling may be one of the mechanisms underlying neurodegenerative disease that causes progressive impairment of learning, memory, and cognitive functions.

A previous randomized controlled trial has reported that patients with diabetes have worse cognitive performance than patients without diabetes; however, whether the incidence of dementia or cognitive impairment in patients with DM could benefit from glycemic control remains controversial (Moore et al., 2013; Biessels et al., 2021). The aim of this meta-analysis was therefore to investigate whether glycemic control in patients with DM or hyperglycemia can delay the degree of decline according to cognitive function assessment scores.

## 2. Materials and methods

### 2.1. For protocol registration

This systematic review was registered on the PROSPERO International prospective register of systematic reviews (CRD42022342260).

### 2.2. Search methods

We searched four medical databases, PubMed, EMBASE, Cochrane Library, and the clinical registry ClinicalTrials.gov, for studies published from database inception to June 2022. Terms used as subject headings in the search strategy included cognitive impairment, dementia, blood glucose, hyperglycemia, antidiabetic drugs, insulin resistance, and randomized controlled trials. Please see the supplemental information for the complete search strategy. There were no restrictions on the language or country of publication.

### 2.3. Inclusion and exclusion criteria

Randomized controlled trials assessing changes in cognitive function in patients with DM, hyperglycemia, or insulin resistance treated with controls or antidiabetic drugs, and who underwent follow-up for at least 3 months with reported cognition scores were screened and finally enrolled. The experimental group was treated with antidiabetic drugs while the control group was treated with placebo or another active antidiabetic drug (Table 1).

**Table 1:** The clinical characteristics of enrolled studies.

Study	Country	Trial design	Sample size	Age (Mean)	Sex (Male/Female)	Intervention		Cognitive assessment	Cognitive score (Mean)		Follow-up (Month)
						Ex	Con		Baseline	Endpoint	
Biessels et al. (2021)	Netherlands	Randomized, double-blind, active-controlled	Ex: 1618 Con: 1545	Ex: 64.4 Con: 64.4	Ex:1002/616 Con:958/587	Linaliptin	Glimepiride	MMSE	Ex: 28.5 Con: 28.5	Ex: 28.2 Con: 28.3	40
Cukierman-Yaffe et al. (2014)	40 countries	Multicentre randomized open-label	Ex: 1683 Con: 1709	Ex: 62.71 Con: 62.84	Ex:1066/617 Con:940/769	Insulin glargine	Standard care	MMSE	Ex: 27.93 Con: 27.88	Ex: 27.65 Con: 27.36	74
Cukierman-Yaffe et al. (2020)	24 countries	Randomized, double-blind, placebo-controlled	Ex: 4351 Con: 4245	Ex: 65.5 Con: 65.5	Ex:2306/2045 Con:2292/1953	Dulaglutide	Placebo	MoCA	Ex: 25 Con: 25	Ex: 24.54 Con: 24.47	24
Cummings et al. (2021)	13 countries	Randomized, double-blind, active-controlled	Ex: 64 Con: 80	Ex: 73 Con: 73.5	Ex:45/19 Con:53/27	Apabetalone	Placebo	MoCA	Ex: 24 Con: 24	Ex: 24.5 Con: 24.4	12
Furie et al. (2018)	United States	Randomized, double-blind, placebo- controlled	Ex: 1699 Con: 1699	Ex: 63.0 Con: 63.1	Ex: 1140/559 Con:1113/586	Pioglitazone	Placebo	3MS	Ex: 96 Con: 97	Ex: 95.9 Con: 96.7	60
Guo et al., 2014	China	Randomized, double-blind, active-controlled	Ex: 29 Con: 29	Ex: 54.7 Con: 53.3	Ex:17/12 Con:19/10	Metformin	Placebo	WMS-R	Ex: 78.6 Con: 77.7	Ex: 99.2 Con: 77.1	6
Hanyu et al. (2009)	Japan	Prospective randomized, open-controlled	Ex: 15 Con: 17	Ex: 56.3 Con: 55.9	Ex:7/8 Con:8/9	Pioglitazone	Placebo	MMSE	Ex: 22.2 Con: 22.4	Ex: 23.1 Con: 22.1	6
Köbe et al., 2017	Germany	Randomized, double-blind, interventional	Ex: 18 Con: 22	Ex: 65 Con: 69	Ex: 8/10 Con:11/11	Resveratrol	Placebo	AVLT subtests	Ex: 44.9 Con: 44.2	Ex: 43.0 Con: 41.9	6.5
Li et al. (2021)	China	Prospective parallel, open-label	Ex: 24 Con: 23	Ex: 55.0 Con: 59.5	Ex:14/10 Con:9/14	GLP-1	Oral antidiabetic drugs	MMSE	Ex: 27.92 Con: 27.39	Ex: 28.96 Con: 27.48	3
Isik et al. (2017)	Turkey	Prospective, observational	Ex: 104 Con: 101	Ex: 74.75 Con: 76.12	Ex:47/57 Con:35/66	Sitagliptin +M39	Placebo	MMSE	Ex: 23.48 Con: 23.12	Ex: 24.18 Con: 23.12	6
Sato et al. (2011)	Japan	Prospective randomized, open-controlled	Ex: 21 Con: 21	Ex: 77.4 Con: 77.6	Ex:11/10 Con:9/12	Pioglitazone	Placebo	MMSE	Ex: 22.1 Con: 21.9	Ex: 23.1 Con: 21.6	6
Plastino et al. (2010)	Italy	Prospective, open label, observational study	Ex: 55 Con: 49	Ex: 81.7 Con: 73.7	Ex:26/29 Con:23/26	insulin+oral antidiabetic medication	Oral antidiabetic medication	MMSE	Ex: 21.9 Con: 20.4	Ex: 21.7 Con: 19.8	6
Lin et al. (2018)	China	Randomized, double-blind, placebo-controlled	Ex: 48 Con: 46	Ex: 66.5 Con: 67.4	Ex:26/22 Con:27/19	Metformin	Acarbose	AVLT	Ex: 16.1 Con: 15.9	Ex: 17.9 Con: 15.6	12

Ex, experimental; Con, control.

Studies with incomplete information or where the full text was not available were excluded. For duplicate studies, the most recent publications were selected. We further excluded reviews, retrospective studies, case reports, animal studies, and unrelated studies.

### 2.4. Outcomes

The primary outcome indicators for the cognitive function assessment were the Mini-Mental State Examination (MMSE) scale, Montreal Cognitive Assessment (MoCA) scale, Modified Mini-Mental State (3MS) scale, Wechsler Memory Scale Revised (WMS-R), and Auditory Word Learning Test (AVLT). In addition, the digit symbol substitution test (DSST) was selected as a secondary outcome indicator.

### 2.5. Study selection and data extraction

The Endnote X9 software was used for literature management. Two researchers (Yufeng Lin and Kaiyuan Wang) searched and downloaded literature according to the search strategy, and deleted any duplicates. Any disagreements were resolved by discussion with a third researcher (Zhongying Gong). Two researchers (Yufeng Lin and Chunchao Ma) independently screened the articles while referencing the inclusion criteria, and a third researcher (Kaiyuan Wang) helped resolve any disagreement. Through reading of the study titles, abstracts, and full texts, the final selected literature was identified and the reasons for exclusion of other studies were recorded. Details such as the first author, study type, year of publication, sample size, sex, age, intervention, follow-up time, and cognitive function assessment method used were recorded for each study according to a pre-designed standardized information extraction form.

### 2.6. Risk of bias assessment of included studies

The methodological quality of the included literature was evaluated by two researchers (Yufeng Lin and Zhongying Gong) using the Revised Cochrane Risk of Bias tool (RoB 2.0; Lester-Coll et al., 2006). Specific evaluation components included randomization process, deviation from intended interventions, missing outcome data, measurement of outcomes, and selective reporting of outcomes. By reading the full text, the risk of bias for each domain was judged as high, low, or unclear. If all domains were of low risk, the overall risk of bias was considered low, if at least one domain was of high risk, the overall risk of bias was considered to be high, and if any domain showed unclear risk and there were no high risks present in any domain, the overall risk of bias was determined to be unclear. A third researcher (Chunchao Ma) convened discussions to resolve any disagreement that arose between the two reviewers.

### 2.7. Data synthesis and analysis

The RevMan v5.3 software provided by the Cochrane Collaboration was used to perform statistical analysis of the extracted data. For continuous data, the analysis applied the mean difference (MD) or standardized mean difference (SMD), calculated with 95% confidence intervals (CIs). Cochrane’s X^2^ and I^2^ tests were used to assess heterogeneity. Considering that the different methods of cognitive function assessments used might impact the study results, we conducted subgroup analyzes based on the scoring methods and applied SMD and random effects models for the analysis. To ensure study integrity, we further used the STATA-64 software for sensitivity analysis, and funnel plot analysis was used to detect publication bias.

## 3. Results

### 3.1. Study selection

**Figure 1:**
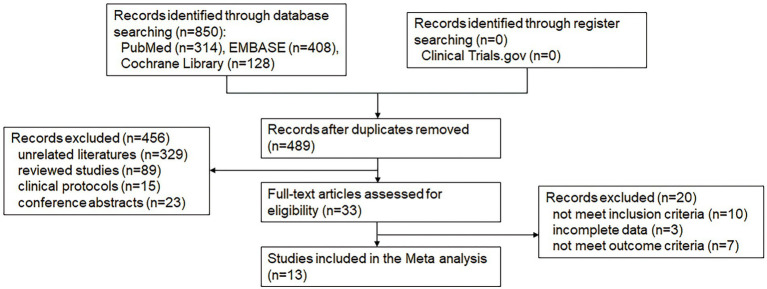
Flow diagram of the study selection process.

A total of 850 studies were retrieved using the search strategy, and 361 duplicate studies were excluded. After screening the retrieved titles and abstracts, 329 irrelevant studies, 89 review studies, 15 clinical study protocols, and 23 congress abstracts were excluded. The remaining 33 full-text studies were retained and evaluated for eligibility. Ten studies that did not meet the inclusion criteria, three studies with incomplete data, and seven studies that did not meet the outcome criteria were excluded. Finally, 13 relevant studies were included (Hanyu et al., 2009; Plastino et al., 2010; Sato et al., 2011; Cukierman-Yaffe et al., 2014, 2020; Guo et al., 2014; Isik et al., 2017; Köbe et al., 2017; Furie et al., 2018; Lin et al., 2018; Biessels et al., 2021; Cummings et al., 2021; Li et al., 2021). The specific literature screening process is shown in Figure 1.

### 3.2. Basic clinical characteristics

Information on the authors, time of publication, country, trial design, sample size, age, intervention modality, cognitive function assessment and scores at enrollment and follow-up are summarized and presented in Table 1.

### 3.3. Risk of bias assessment

**Figure 2:**
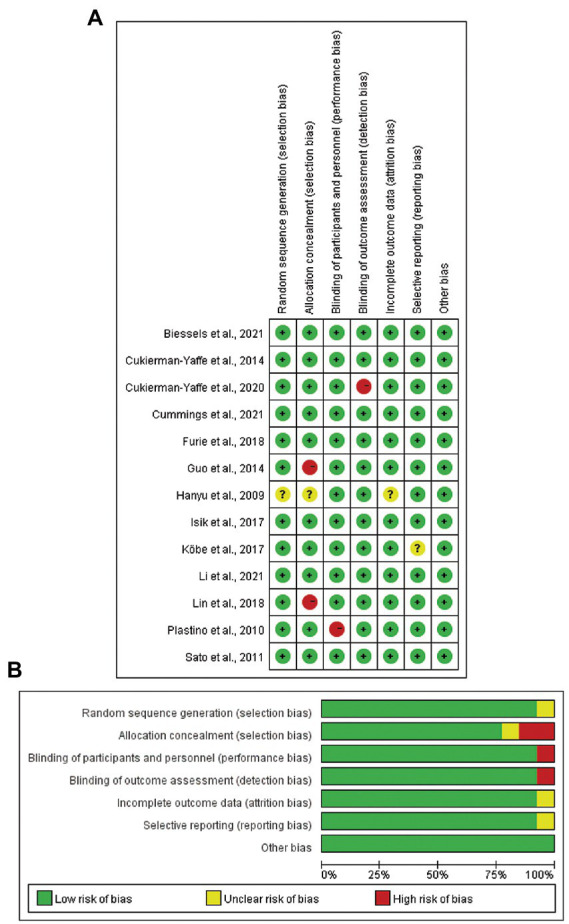
Risk of bias assessment of included studies in the meta-analysis. (**A**) Risk of bias for individual study. (**B**) Overall risk of bias for the 13 included studies.

In terms of risk of bias of individual study, seven studies were classified as low risk (Sato et al., 2011; Cukierman-Yaffe et al., 2014; Isik et al., 2017; Furie et al., 2018; Biessels et al., 2021; Cummings et al., 2021; Li et al., 2021), two studies were moderate risk (Hanyu et al., 2009; Köbe et al., 2017), and four studies were high risk (Plastino et al., 2010; Guo et al., 2014; Lin et al., 2018; Cukierman-Yaffe et al., 2020). Two of the studies (Guo et al., 2014; Lin et al., 2018) did not provide the complete method of allocation concealment (Figure 2A). In terms of the overall risk of bias, there was a low risk of other biases; unclear risks for random sequence generation, incomplete outcome data, and selective reporting; and high risks for allocation concealment, binding of participants and personnel, and binding of outcome assessment (Figure 2B).

### 3.4. Cognitive function assessments

**Figure 3:**
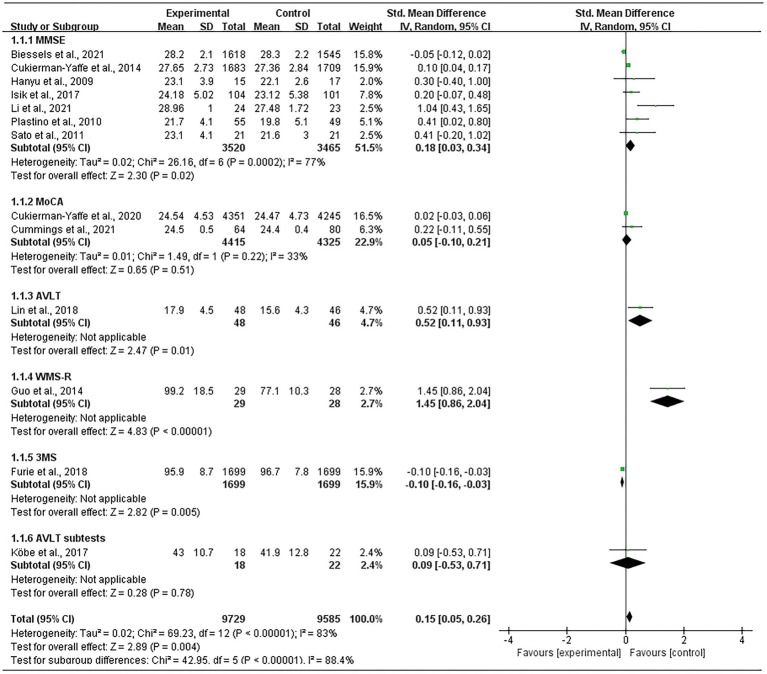
Forest plot of unadjusted standard mean difference in cognitive assessment scores.

Thirteen studies comprising 19,314 participants were included. Analysis revealed that glycemic control significantly attenuated the degree of decline in cognitive function assessment scores (SMD = 0.15; 95% CI 0.05, 0.26; *p* < 0.00001; Figure 3).

#### 3.4.1. Mini-mental state examination

**Figure 4:**
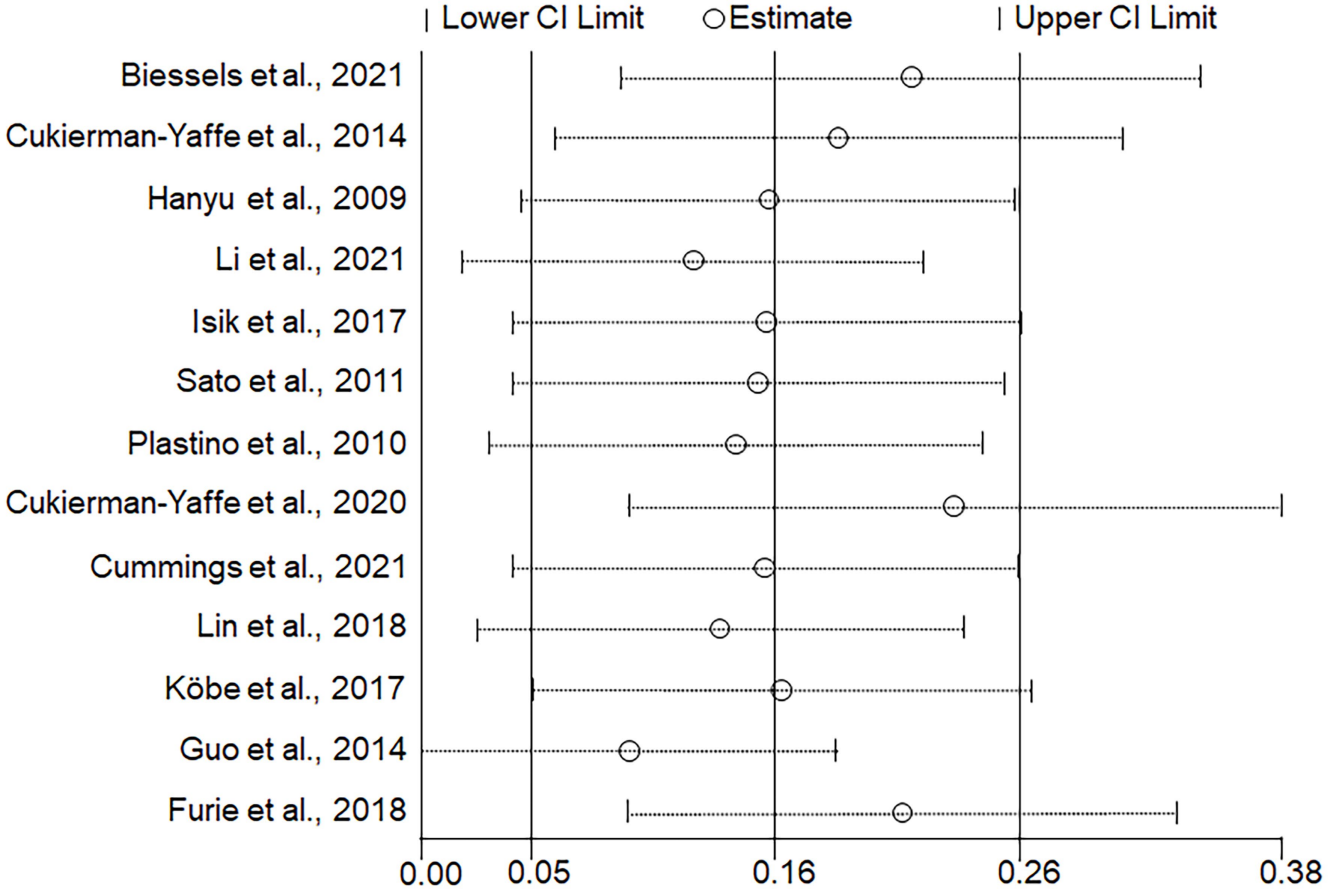
Sensitivity analysis for included studies.

**Figure 5:**
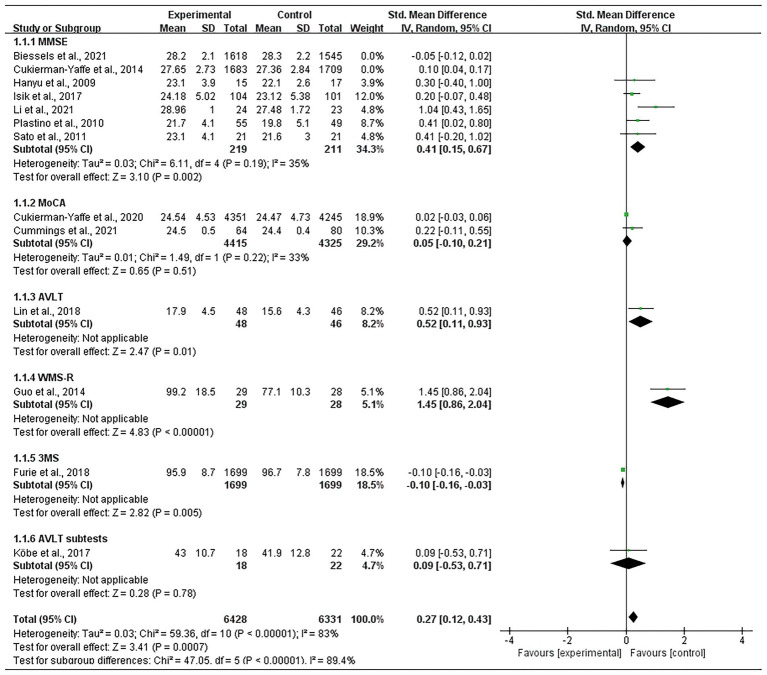
Forest plot of adjusted standard mean difference in cognitive assessment scores.

Seven studies that included a total of 6,985 participants (Hanyu et al., 2009; Plastino et al., 2010; Sato et al., 2011; Cukierman-Yaffe et al., 2014; Isik et al., 2017; Biessels et al., 2021; Li et al., 2021) used MMSE to assess cognitive function. Meta-analysis of these studies was performed using a random effects model, which showed that glycemic control had a significant effect on cognitive function improvement (SMD = 0.18; 95% CI 0.03, 0.34; *p* = 0.02); however, within-group heterogeneity was significant (*p* = 0.0002, I^2^ = 77%; Figure 3). The ReVman software was subsequently used to further examine each study, and the Stata software was used to perform sensitivity analysis (Figure 4). The results indicated that the source of heterogeneity originated from mainly two studies (Cukierman-Yaffe et al., 2014; Biessels et al., 2021). After removing the two, meta-analysis was performed using a fixed response model with the five remaining studies comprising 430 participants, showing that glycemic control remained significant in improving cognitive function (SMD = 0.41; 95% CI 0.15, 0.67; *p* = 0.002). Within-group heterogeneity was within the normal limits (*p* = 0.19, I^2^ = 35%; Figure 5).

#### 3.4.2. Montreal cognitive assessment

Two studies (Cukierman-Yaffe et al., 2020; Cummings et al., 2021) with 8,740 participants used MoCA to assess cognitive function. A meta-analysis of these studies was performed using a random effects model, which showed that glycemic control improved cognitive function, but the results were not significant (SMD = 0.05; 95% CI-0.10, 0.21; *p* = 0.51). The within-group heterogeneity was within the normal range (*p* = 0.22, *I*^2^ = 33%; Figure 5).

#### 3.4.3. Auditory word learning test

**Figure 6:**
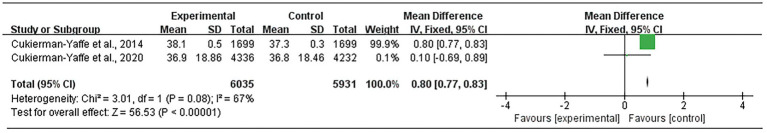
Meta-analysis of DSST by glycemic control.

One study (Lin et al., 2018) which included 94 participants used AVLT for cognitive function assessment. Meta-analysis using a random effects model showed a significant improvement in cognitive function by controlling blood glucose (SMD = 0.52; 95% CI 0.11, 0.93; *p* = 0.01; Figure 6). Meta-analysis using a random effects model of another study (Köbe et al., 2017) with 40 participants used AVLT subtests for cognitive function assessment and showed an improvement in cognitive function by controlling blood glucose, but without statistical significance (SMD = 0.09; 95% CI-0.53, 0.71, *p* = 0.78; Figure 5).

#### 3.4.4. Wechsler memory scale revised

One study (Guo et al., 2014) included 57 participants and used WMS-R for cognitive function assessment. Meta-analysis using a random effects model showed a significant improvement in cognitive function by controlling blood glucose (SMD = 1.45; 95% CI 0.86, 2.04; *p* < 0.00001; Figure 5).

#### 3.4.5. Modified mini-mental state

One study (Furie et al., 2018) with 3,398 participants used 3MS for cognitive function assessment. Meta-analysis of this study using a random-effects model showed a significant effect of controlling blood glucose on improvement in cognitive function (SMD = -0.10; 95% CI-0.16, −0.03; *p* = 0.005; Figure 5).

#### 3.4.6. Digit symbol substitution test

Two studies (Cukierman-Yaffe et al., 2014, 2020) including 11,966 participants used DSST for secondary assessment of cognitive function. Meta-analysis performed on these studies using a fixed effects model showed that glycemic control had a significant effect on increasing DSST scores (SMD = -0.80; 95% CI 0.77, 0.83, p < 0.00001; Figure 6).

### 3.5. Publication bias

**Figure 7:**
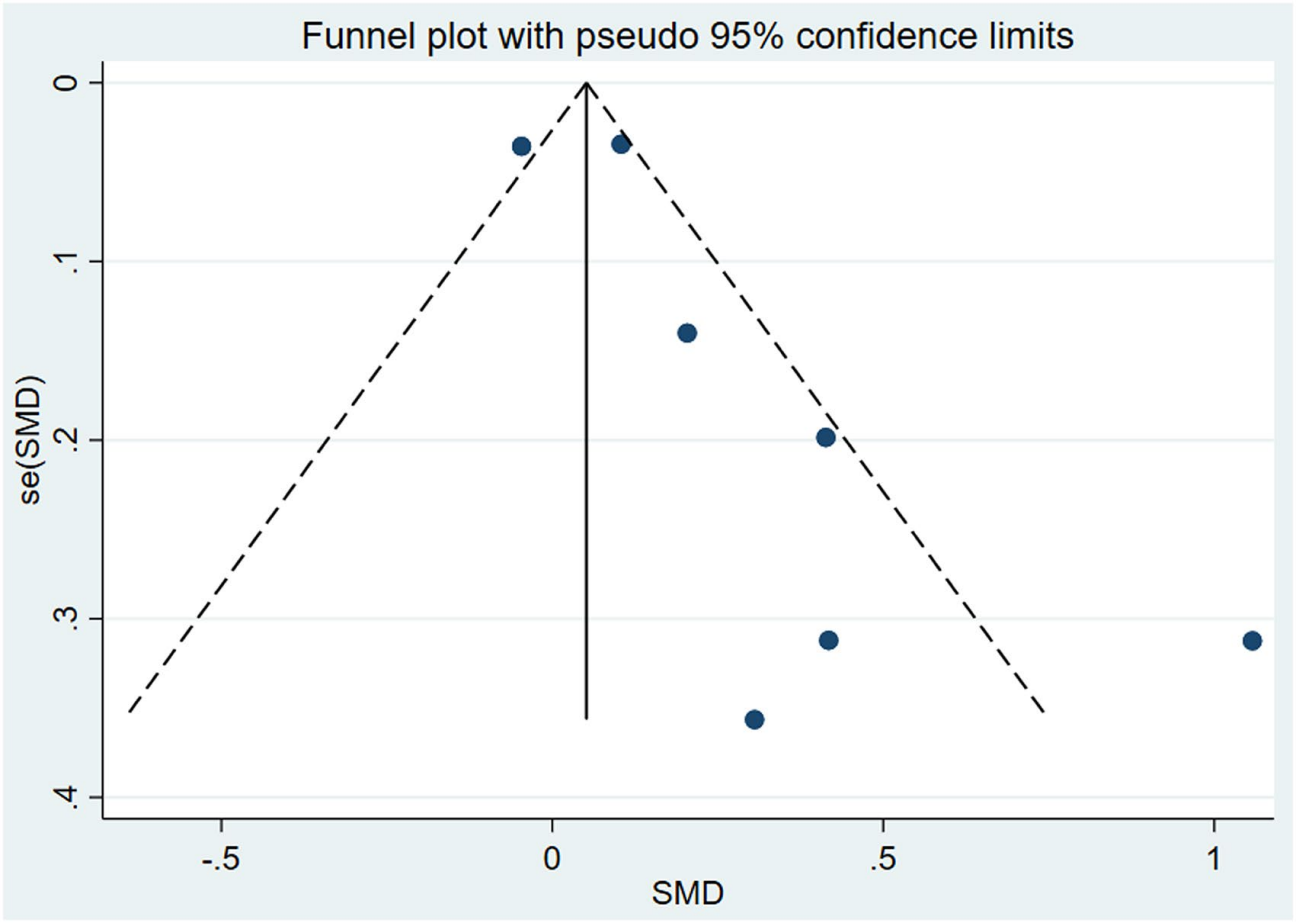
Funnel plot of studies using MMSE as cognitive evaluation.

**Figure 8:**
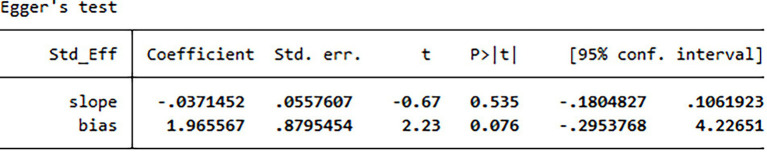
Egger’s test of publication bias.

For the seven studies in which MMSE was the primary assessment method of cognitive function, we performed publication bias analysis and subsequently created funnel plots. As shown in Figure 7, the left and right scatter points within the plot were largely symmetrical, and Egger’s test further confirmed no publication bias (*p* = 0.076; see Figure 8).

## 4. Discussion

The incidence of hyperglycemia or DM and cognitive impairment both increase progressively with age. In a 10-year population-based cohort study of individuals aged 65 years and older, a modest degree of hyperglycemia was proven to independently predispose to faster cognitive decline, and glucose and hemoglobin A1c (HbA1c) were proposed as more sensitive markers of glycemia (Ganguli et al., 2020). Other studies have shown that the risk of developing cognitive decline or dementia in patients with type 2 DM is 1.25 to 2 times higher than that in patients without diabetes (Gudala et al., 2013; Xue et al., 2019). Morris et al. (2016) used the hyperinsulinemic-euglycemic clamp technique to detect systemic insulin resistance in patients with mild cognitive impairment (MCI) and AD as compared to normal controls, observing increased insulin resistance in 15 patients with cognitive impairment. Even in children with newly diagnosed type 1 DM, a single DKA episode was found to be associated with cognitive decline, particularly in subtle memory function (Ghetti et al., 2020). Although severe hypoglycemia may also lead to poor global cognition in older adults (Lacy et al., 2020), mounting clinical evidence has shown that cognitive impairment is exacerbated by hyperglycemia or DM in large populations.

The pathophysiological process of cognitive decline in patients with hyperglycemia or DM is complex and may involve common features with the pathogenesis of AD and vascular dementia (Gerstein et al., 2020), although the molecular interactions between the two diseases are not fully understood. The physio-pathological mechanisms that characterize AD, including molecular, biochemical, and signaling abnormalities, are known to be similar to those of patients with diabetes. In addition, reduced insulin signaling in the brain due to insulin dysfunction may be the primary mechanism shared by both diseases (Duarte et al., 2012). The concept of “insulin-resistant brain state (IRBS)” has thus been proposed to better describe the nature of AD (de la Monte and Wands, 2008). Insulin resistance is associated with reduced cortical insulin receptor activation, impaired clearance of amyloid-β (Aβ) oligomers, increased cerebral abnormal neurotic plaque burden, and the cerebral microvascular dysfunction which is associated with memory loss or decline of cognition (Umegaki et al., 2017; van Sloten et al., 2020). Glucotoxicity from the accumulation of advanced glycation end products (AGEs) and their precursor methylglyoxal (MGO) could induce dopaminergic dysfunction, thereby playing a role in DM-associated cognitive impairment (Pignalosa et al., 2021).

In the present study, we reviewed and evaluated the potential protective effect of blood glucose control therapy on cognitive function in patients with DM, hyperglycemia, or insulin resistance. Thirteen trials with 19,134 participants were enrolled for preliminary outcome analysis. The MMSE, MoCA, AVLT, WMS-R, 3MS, and AVLT were used as primary cognitive function assessment methods. Overall analysis showed that glycemic control significantly attenuated cognitive decline. Several recent reviews and meta-analyzes have also investigated the relationship between antidiabetic therapy and cognitive status, with inconsistent primary findings (Areosa Sastre et al., 2017; Cao et al., 2018; McMillan et al., 2018). These inconsistencies are mainly due to differences in the focus and detailed design of the studies. For example, Areosa Sastre’s review only enrolled patients diagnosed with type 2 DM, while Cao’s study enrolled patients diagnosed with Alzheimer’s disease, but was not restricted to those with DM. In McMillan’s review, only the incidence of dementia was analyzed, and the change in cognitive score which may compromise the potential cerebral protection of blood glucose control therapy was not evaluated (McMillan et al., 2018).

## 5. Limitations

The present review and analysis have several limitations which should be noted. First, the enrolled studies applied different cognitive function assessment methods, resulting in heterogeneity between groups. Second, the studies had various follow-up times, and longer follow-up periods would have allowed for more accurate detection of changes in cognitive function. Third, the optimal glycemic range for the prevention of cognitive decline could not be determined in this study, and thus further exploration through high-quality clinical trials is required.

## 6. Conclusion

In conclusion, the current study provides evidence that glycemic control could improve the cognitive impairment through cognitive function assessment scores in patients with DM, hyperglycemia or insulin resistance.

## Data availability statement

The raw data supporting the conclusions of this article will be made available by the authors, without undue reservation.

## Author contributions

YL, KW, and ZW designed and carried out the study. ZG and CM participated in analyzing and interpretation of the results. YL and KW wrote the manuscript with other authors’ inputs. ZW revised the manuscript. All authors contributed to the article and approved the submitted version.

## Funding

This research was partially funded by grants from Tianjin “project + team” key cultivation program (No. XC202034) and Tianjin Key Medical Discipline (Specialty) Construction Project (No.TJYXZDXK-009A).

## Conflict of interest

The authors declare that the research was conducted in the absence of any commercial or financial relationships that could be construed as a potential conflict of interest.

The handling editor XG declared a shared parent affiliation with the author KW at the time of review.

## Publisher’s note

All claims expressed in this article are solely those of the authors and do not necessarily represent those of their affiliated organizations, or those of the publisher, the editors and the reviewers. Any product that may be evaluated in this article, or claim that may be made by its manufacturer, is not guaranteed or endorsed by the publisher.
