# Supplementary material for: Relationship between glycemic control and cognitive impairment: A systematic review and meta-analysis
Source: Front Aging Neurosci. 2023 Jan 26;15:1126183. doi: 10.3389/fnagi.2023.1126183 (PMC9909073; doi:10.3389/fnagi.2023.1126183)
Supplement: Supplementary file 1 [file Table_1.DOCX]

| **The search strategy for PubMed** | |
| --- | --- |
| **Nubmer** | **Search terms** |
| #1 | **Cognitive Dysfunction**[MeSH Terms] |
| #2 | (((((((((((((((((((((((((((((Cognitive Dysfunction[Title/Abstract]) OR Cognitive Dysfunctions[Title/Abstract]) OR Dysfunction, Cognitive[Title/Abstract]) OR Dysfunctions, Cognitive[Title/Abstract]) OR Cognitive Impairments[Title/Abstract]) OR Cognitive Impairment[Title/Abstract]) OR Impairment, Cognitive[Title/Abstract]) OR Impairments, Cognitive[Title/Abstract]) OR Mild Cognitive Impairment[Title/Abstract]) OR Cognitive Impairment, Mild[Title/Abstract]) OR Cognitive Impairments, Mild[Title/Abstract]) OR Impairment, Mild Cognitive[Title/Abstract]) OR Impairments, Mild Cognitive[Title/Abstract]) OR Mild Cognitive Impairments[Title/Abstract]) OR Mild Neurocognitive DisORder[Title/Abstract]) OR DisORder, Mild Neurocognitive[Title/Abstract]) OR DisORders, Mild Neurocognitive[Title/Abstract]) OR Mild Neurocognitive DisORders[Title/Abstract]) OR Neurocognitive DisORder, Mild[Title/Abstract]) OR Neurocognitive DisORders[Title/Abstract]) OR Mild Cognitive Decline[Title/Abstract]) OR Cognitive Declines[Title/Abstract]) OR Decline, Cognitive[Title/Abstract]) OR Declines, Cognitive[Title/Abstract]) OR Mental DeteriORation[Title/Abstract]) OR DeteriORation, Mental[Title/Abstract]) OR DeteriORations, Mental[Title/Abstract]) OR Mental DeteriORations[Title/Abstract]))) |
| #3 | **Dementia, Vascular**[MeSH Terms] |
| #4 | (((((((((((((((((((((((((((((((((((((((((Dementia, Vascular[Title/Abstract]) OR Dementias, Vascular[Title/Abstract]) OR Vascular Dementias[Title/Abstract]) OR Vascular Dementia[Title/Abstract]) OR Vascular Dementia, Acute Onset[Title/Abstract]) ORAcute Onset Vascular Dementia[Title/Abstract]) OR SubcORtical Vascular Dementia[Title/Abstract]) OR Dementia, SubcORtical Vascular[Title/Abstract]) OR Dementias, SubcORtical Vascular[Title/Abstract]) OR SubcORtical Vascular Dementias[Title/Abstract]) OR Vascular Dementia, SubcORtical[Title/Abstract]) OR Vascular Dementias, SubcORtical[Title/Abstract]) OR Arteriosclerotic Dementia[Title/Abstract]) OR Arteriosclerotic Dementias[Title/Abstract]) OR Dementia, Arteriosclerotic[Title/Abstract]) OR Dementias, Arteriosclerotic[Title/Abstract]) OR Binswanger Disease[Title/Abstract]) OR Disease, Binswanger[Title/Abstract]) OR Chronic Progressive SubcORtical Encephalopathy[Title/Abstract]) OR Binswanger Encephalopathy[Title/Abstract]) OR Leukoencephalopathy, SubcORtical[Title/Abstract]) OR Leukoencephalopathies, SubcORtical[Title/Abstract]) OR SubcORtical Leukoencephalopathies[Title/Abstract]) OR Encephalopathy, SubcORtical Arteriosclerotic[Title/Abstract]) OR Binswanger's Disease[Title/Abstract]) OR Binswangers Disease[Title/Abstract]) OR Disease, Binswanger's[Title/Abstract]) OR Encephalopathy, SubcORtical, Chronic Progressive[Title/Abstract]) OR SubcORtical Encephalopathy, Chronic Progressive[Title/Abstract]) OR SubcORtical Leukoencephalopathy[Title/Abstract]) OR SubcORtical Arteriosclerotic Encephalopathy[Title/Abstract]) OR Arteriosclerotic Encephalopathy, SubcORtical[Title/Abstract]) OR Arteriosclerotic Encephalopathies, SubcORtical[Title/Abstract]) OR Encephalopathies, SubcORtical Arteriosclerotic[Title/Abstract]) OR SubcORtical Arteriosclerotic Encephalopathies[Title/Abstract]) OR Encephalopathy, Binswanger's[Title/Abstract]) OR Binswanger's Encephalopathy[Title/Abstract]) OR Encephalopathy, Binswangers[Title/Abstract]) OR Encephalopathy, Binswanger[Title/Abstract]) OR Encephalopathy, Chronic Progressive SubcORtical[Title/Abstract]))) |
| #5 | Dementia, Multi-Infarct[MeSH Terms] |
| #6 | (((((((((((((((((((Dementia, Multi-Infarct[Title/Abstract]) OR Dementia, Multi Infarct[Title/Abstract]) OR Dementias, Multi-Infarct[Title/Abstract]) OR Multi-Infarct Dementias[Title/Abstract]) OR Dementia Multi-Infarct[Title/Abstract]) OR Dementia Multi Infarct[Title/Abstract]) OR Dementia Multi-Infarcts[Title/Abstract]) OR Multi-Infarct, Dementia[Title/Abstract]) OR Multi-Infarcts, Dementia[Title/Abstract]) OR Dementia, Multiinfarct[Title/Abstract]) OR Dementias, Multiinfarct[Title/Abstract]) OR Multiinfarct Dementia[Title/Abstract]) OR Multiinfarct Dementias[Title/Abstract]) OR Multi-Infarct Dementia[Title/Abstract]) OR Multi Infarct Dementia[Title/Abstract]) OR Lacunar Dementia[Title/Abstract]) OR Dementia, Lacunar[Title/Abstract]) OR Dementias, Lacunar[Title/Abstract]) OR Lacunar Dementias[Title/Abstract])) OR ((((((((PSCI[Title/Abstract]) OR PSD[Title/Abstract]) OR PSCIND[Title/Abstract]) OR post stroke cognitive impairment[Title/Abstract]) OR cognitive impairment after stroke[Title/Abstract]) OR post stroke cognitive impairment no dementia[Title/Abstract]) OR post stroke dementia[Title/Abstract]))) |
| #7 | #1 OR #2 OR #3 OR #4 OR #5 OR #6 |
| #8 | Hyperglycemia[MeSH Terms] |
| #9 | (((((Hyperglycemias[Title/Abstract]) OR (Hyperglycemia, Postprandial[Title/Abstract])) OR (Hyperglycemias, Postprandial[Title/Abstract])) OR (Postprandial Hyperglycemias[Title/Abstract])) OR (Postprandial Hyperglycemia[Title/Abstract]))) |
| #10 | **Blood Glucose**[MeSH Terms] |
| #11 | (((Blood Sugar[Title/Abstract]) OR (Sugar, Blood[Title/Abstract])) OR (Glucose, Blood[Title/Abstract]))) |
| #12 | Hypoglycemic Agents[MeSH Terms] |
| #13 | (((((((((((((((((((((((((((((Agents, Hypoglycemic[Title/Abstract]) OR (Hypoglycemic Agent[Title/Abstract])) OR (Agent, Hypoglycemic[Title/Abstract])) OR (Antihyperglycemic Agent[Title/Abstract])) OR (Agent, Antihyperglycemic[Title/Abstract])) OR (Antihyperglycemics[Title/Abstract])) OR (Hypoglycemic[Title/Abstract])) OR (Hypoglycemic Drugs[Title/Abstract])) OR (Drugs, Hypoglycemic[Title/Abstract])) OR (Hypoglycemics[Title/Abstract])) OR (Antihyperglycemic Agents[Title/Abstract])) OR (Agents, Antihyperglycemic[Title/Abstract])) OR (Antihyperglycemic[Title/Abstract])) OR (Hypoglycemic Drug[Title/Abstract])) OR (Drug, Hypoglycemic[Title/Abstract])) OR (Antidiabetics[Title/Abstract])) OR (Antidiabetic Drug[Title/Abstract])) OR (Drug, Antidiabetic[Title/Abstract])) OR (Antidiabetic Drugs[Title/Abstract])) OR (Drugs, Antidiabetic[Title/Abstract])) OR (Antidiabetic Agents[Title/Abstract])) OR (Agents, Antidiabetic[Title/Abstract])) OR (Antidiabetic Agent[Title/Abstract])) OR (Agent, Antidiabetic[Title/Abstract])) OR (Antidiabetic[Title/Abstract])) OR (Hypoglycemic Effect[Title/Abstract])) OR (Effect, Hypoglycemic[Title/Abstract])) OR (Hypoglycemic Effects[Title/Abstract])) OR (Effects, Hypoglycemic[Title/Abstract])) |
| #14 | Insulin Resistance[MeSH Terms] |
| #15 | (((Resistance, Insulin[Title/Abstract]) OR (Insulin Sensitivity[Title/Abstract])) OR (Sensitivity, Insulin[Title/Abstract]))) |
| #16 | Glucose Metabolism Disorders[MeSH Terms] |
| #17 | (((((((((((Disorder, Glucose Metabolism[Title/Abstract]) OR (Disorders, Glucose Metabolism[Title/Abstract])) OR (Metabolism Disorder, Glucose[Title/Abstract])) OR (Metabolism Disorders, Glucose[Title/Abstract])) OR (Glucose Metabolic Disorders[Title/Abstract])) OR (Disorder, Glucose Metabolic[Title/Abstract])) OR (Disorders, Glucose Metabolic[Title/Abstract])) OR (Metabolic Disorder, Glucose[Title/Abstract])) OR (Metabolic Disorders, Glucose[Title/Abstract])) OR (Glucose Metabolism Disorder[Title/Abstract])) OR (Glucose Metabolic Disorder[Title/Abstract]))) or Abnormal Glucose Metabolism)) |
| #18 | #8 OR #9 OR #10 OR #11 OR #12 OR #13 OR #14 OR #15 OR #16 OR #17 |
| #19 | ((randomized controlled trial[Publication Type] OR randomized[Title/Abstract] OR placebo[Title/Abstract]) OR randomly[Title/Abstract]) |
| #20 | #7 AND #18 AND #19 |
| **The search strategy for Cochrane library** | |
| **Nubmer** | **Search terms** |
| #1 | MeSH descriptor: [Stroke] explode all trees |
| #2 | (Stroke*):ti,ab,kw OR (Cerebrovascular Accident*):ti,ab,kw OR (CVA*):ti,ab,kw OR (Apoplexy, Cerebrovascular):ti,ab,kw OR (Vascular Accident*, Brain):ti,ab,kw OR (Cerebrovascular Stroke*):ti,ab,kw OR (Stroke*, Cerebrovascular):ti,ab,kw OR (Apoplexy):ti,ab,kw OR (Cerebral Stroke*):ti,ab,kw OR (Stroke*, Cerebral):ti,ab,kw OR (Stroke*, Acute):ti,ab,kw OR (Acute Stroke*):ti,ab,kw OR (Cerebrovascular Accident*, Acute):ti,ab,kw OR (Acute Cerebrovascular Accident*):ti,ab,kw |
| #3 | MeSH descriptor: [Cerebral HemORrhage] explode all trees |
| #4 | (HemORrhage*, Cerebrum):ti,ab,kw OR (Cerebrum HemORrhage*):ti,ab,kw OR (Parenchymal HemORrhage*, Cerebral):ti,ab,kw OR (Intracerebral HemORrhage*):ti,ab,kw OR (HemORrhage*, Intracerebral):ti,ab,kw OR (HemORrhage*, Cerebral):ti,ab,kw OR (Cerebral HemORrhage*):ti,ab,kw OR (Brain HemORrhage*, Cerebral):ti,ab,kw |
| #5 | #1OR #2 OR #3 OR #4 |
| #6 | MeSH descriptor: [Cognitive Dysfunction] explode all trees |
| #7 | (Dysfunctions, Cognitive):ti,ab,kw OR (Cognitive Impairments):ti,ab,kw OR (Impairments, Cognitive):ti,ab,kw OR (Impairment, Cognitive):ti,ab,kw OR (Dysfunction, Cognitive):ti,ab,kw OR (Cognitive Dysfunctions):ti,ab,kw OR (Cognitive Impairment):ti,ab,kw OR (Mild Neurocognitive DisORders):ti,ab,kw OR (Impairment, Mild Cognitive):ti,ab,kw OR (Neurocognitive DisORders, Mild):ti,ab,kw OR (Mild Cognitive Impairment):ti,ab,kw OR (Impairments, Mild Cognitive):ti,ab,kw OR (DisORder, Mild Neurocognitive):ti,ab,kw OR (Mild Cognitive Impairments):ti,ab,kw OR (Cognitive Impairment, Mild):ti,ab,kw OR (Mild Neurocognitive DisORder):ti,ab,kw OR (DisORders, Mild Neurocognitive):ti,ab,kw OR (Neurocognitive DisORder, Mild):ti,ab,kw OR (Cognitive Impairments, Mild):ti,ab,kw OR (Mental DeteriORations):ti,ab,kw OR (Decline, Cognitive):ti,ab,kw OR (Cognitive Decline):ti,ab,kw OR (Mental DeteriORation):ti,ab,kw OR (DeteriORations, Mental):ti,ab,kw OR (DeteriORation, Mental):ti,ab,kw OR (Declines, Cognitive):ti,ab,kw OR (Cognitive Declines):ti,ab,kw |
| #8 | MeSH descriptor: [Dementia, Vascular] explode all trees |
| #9 | (Vascular Dementias):ti,ab,kw OR (Dementias, Vascular):ti,ab,kw OR (Vascular Dementia):ti,ab,kw OR (Dementia, SubcORtical Vascular):ti,ab,kw OR (Vascular Dementia, SubcORtical):ti,ab,kw OR (Dementias, SubcORtical Vascular):ti,ab,kw OR (SubcORtical Vascular Dementias):ti,ab,kw OR (Vascular Dementias, SubcORtical):ti,ab,kw OR (SubcORtical Vascular Dementia):ti,ab,kw OR (Binswanger's Encephalopathy):ti,ab,kw OR (Binswanger Encephalopathy):ti,ab,kw OR (Chronic Progressive SubcORtical Encephalopathy):ti,ab,kw OR (Encephalopathy, Binswangers):ti,ab,kw OR (Arteriosclerotic Encephalopathies, SubcORtical):ti,ab,kw OR (Disease, Binswanger):ti,ab,kw OR (Arteriosclerotic Encephalopathy, SubcORtical):ti,ab,kw OR (Encephalopathies, SubcORtical Arteriosclerotic):ti,ab,kw OR (SubcORtical Arteriosclerotic Encephalopathies):ti,ab,kw OR (SubcORtical Arteriosclerotic Encephalopathy):ti,ab,kw OR ( Encephalopathy, SubcORtical, Chronic Progressive):ti,ab,kw OR (Leukoencephalopathies, SubcORtical):ti,ab,kw OR (SubcORtical Leukoencephalopathies):ti,ab,kw OR (Encephalopathy, Binswanger's):ti,ab,kw OR (Encephalopathy, Chronic Progressive SubcORtical):ti,ab,kw OR (Disease, Binswanger's):ti,ab,kw OR (Encephalopathy, SubcORtical Arteriosclerotic):ti,ab,kw OR ( Leukoencephalopathy, SubcORtical):ti,ab,kw OR ( SubcORtical Leukoencephalopathy):ti,ab,kw OR (Binswangers Disease):ti,ab,kw OR (SubcORtical Encephalopathy, Chronic Progressive):ti,ab,kw OR (Binswanger's Disease):ti,ab,kw OR (Binswanger Disease):ti,ab,kw OR (Encephalopathy, Binswanger):ti,ab,kw OR (Acute Onset Vascular Dementia):ti,ab,kw OR (Vascular Dementia, Acute Onset):ti,ab,kw OR (Arteriosclerotic Dementias):ti,ab,kw OR (Dementia, Arteriosclerotic):ti,ab,kw OR (Arteriosclerotic Dementia):ti,ab,kw OR (Dementias, Arteriosclerotic):ti,ab,kw |
| #10 | MeSH descriptor: [Dementia, Multi-Infarct] explode all trees |
| #11 | (Multi-Infarct Dementias):ti,ab,kw OR (Multi Infarct Dementia):ti,ab,kw OR (Multi-Infarct, Dementia):ti,ab,kw OR (Multiinfarct Dementia):ti,ab,kw OR (Multi-Infarct Dementia):ti,ab,kw OR (Dementia Multi-Infarcts):ti,ab,kw OR (Dementia, Multi Infarct):ti,ab,kw OR (Dementias, Multi-Infarct):ti,ab,kw OR (Multi-Infarcts, Dementia):ti,ab,kw OR (Dementia, Multiinfarct):ti,ab,kw OR (Dementia Multi-Infarct):ti,ab,kw OR (Dementia Multi Infarct):ti,ab,kw OR (Dementias, Multiinfarct):ti,ab,kw OR (Multiinfarct Dementias):ti,ab,kw OR (Dementia, Lacunar):ti,ab,kw OR (Dementias, Lacunar):ti,ab,kw OR (Lacunar Dementia):ti,ab,kw OR (Lacunar Dementias):ti,ab,kw |
| #12 | (PSCI):ti,ab,kw OR(PSD):ti,ab,kw OR(PSCIND):ti,ab,kw OR(post stroke cognitive impairment):ti,ab,kw OR(cognitive impairment after stroke):ti,ab,kw OR(post stroke cognitive impairment no dementia):ti,ab,kw OR(post stroke dementia):ti,ab,kw |
| #13 | #6 OR #7 OR #8 OR #9 OR #10 OR #11 OR #12 |
| #14 | MeSH descriptor:[hyperglycemia] explode all trees |
| #15 | (hyperglycemias): ti,ab,kw OR (hyperglycemia, postprandial): ti,ab,kw OR (hyperglycemias, postprandial): ti,ab,kw OR (postprandial hyperglycemias):ti,ab,kw OR (postprandial hyperglycemia): ti,ab,kw |
| #16 | MeSH descriptor:[blood AND glucose] explode all trees |
| #17 | (blood sugar):ti,ab,kw OR (sugar, blood):ti,ab,kw OR (glucose, blood) :ti,ab,kw |
| #18 | MeSH descriptor:[ hypoglycemic AND agents] explode all trees |
| #19 | (hypoglycemic):ti,ab,kw OR (hypoglycemic agent):ti,ab,kw OR (agent, hypoglycemic):ti,ab,kw OR (antihyperglycemic agent):ti,ab,kw OR (agent, antihyperglycemic):ti,ab,kw OR (antihyperglycemics):ti,ab,kw OR  (hypoglycemic):ti,ab,kw OR (hypoglycemic drugs):ti,ab,kw OR (drugs, hypoglycemic):ti,ab,kw OR (hypoglycemics):ti,ab,kw OR (antihyperglycemic agents):ti,ab,kw OR (agents, antihyperglycemic):ti,ab,kw OR (antihyperglycemic):ti,ab,kw OR (hypoglycemic drug):ti,ab,kw OR (drug, hypoglycemic):ti,ab,kw OR (antidiabetics):ti,ab,kw OR (antidiabetic drug):ti,ab,kw OR (drug, antidiabetic):ti,ab,kw OR (antidiabetic drugs):ti,ab,kw OR (drugs, antidiabetic):ti,ab,kw OR (antidiabetic agents):ti,ab,kw OR (agents, antidiabetic):ti,ab,kw OR (antidiabetic agen):ti,ab,kw OR (agent, antidiabetic):ti,ab,kw OR (antidiabetic):ti,ab,kw OR (hypoglycemic effect):ti,ab,kw OR (effect, hypoglycemic):ti,ab,kw OR (hypoglycemic  effects):ti,ab,kw OR (effects, hypoglycemic):ti,ab,kw |
| #20 | #14 OR #15 OR #16 OR #17 OR #18 OR #19 |
| #21 | (randomized controlled trial)ti,ab,kw OR (randomized)ti,ab,kw OR (placebo)ti,ab,kw |
| #22 | #13 AND #20 #21 |
| **The search strategy for Embase** | |
| **Nubmer** | **Search terms** |
| #1 | cognitive AND dysfunction |
| #2 | 'cognitive dysfunctions':ab,ti OR 'dysfunction, cognitive':ab,ti OR 'dysfunctions, cognitive':ab,ti OR 'cognitive impairments':ab,ti OR 'cognitive impairment':ab,ti OR 'impairment, cognitive':ab,ti OR 'impairments, cognitive':ab,ti OR 'mild cognitive impairment':ab,ti OR 'cognitive impairment, mild':ab,ti OR 'cognitive impairments, mild':ab,ti OR 'impairment, mild cognitive':ab,ti OR 'impairments, mild cognitive':ab,ti OR 'mild cognitive impairments':ab,ti OR 'mild neurocognitive disorder':ab,ti OR 'disorder, mild neurocognitive':ab,ti OR 'disorders, mild neurocognitive':ab,ti OR 'mild neurocognitive disorders':ab,ti OR 'neurocognitive disorder, mild':ab,ti OR 'neurocognitive disorders, mild':ab,ti OR 'cognitive decline':ab,ti OR 'cognitive declines':ab,ti OR 'decline, cognitive':ab,ti OR 'declines, cognitive':ab,ti OR 'mental deterioration':ab,ti OR 'deterioration, mental':ab,ti OR 'deteriorations, mental':ab,ti OR 'mental deteriorations':ab,ti |
| #3 | 'dementia, vascular'/exp OR 'dementia, vascular' OR (('dementia,'/exp OR dementia,) AND vascular) |
| #4 | 'dementias, vascular':ab,ti OR 'vascular dementias':ab,ti OR 'vascular dementia':ab,ti OR 'vascular dementia, acute onset':ab,ti OR 'acute onset vascular dementia':ab,ti OR 'subcortical vascular dementia':ab,ti OR 'dementia, subcortical vascular':ab,ti OR 'dementias, subcortical vascular':ab,ti OR 'subcortical vascular dementias':ab,ti OR 'vascular dementia, subcortical':ab,ti OR 'vascular dementias, subcortical':ab,ti OR 'arteriosclerotic dementia':ab,ti OR 'arteriosclerotic dementias':ab,ti OR 'dementia, arteriosclerotic':ab,ti OR 'dementias, arteriosclerotic':ab,ti OR 'binswanger disease':ab,ti OR 'disease, binswanger':ab,ti OR 'chronic progressive subcortical encephalopathy':ab,ti OR 'binswanger encephalopathy':ab,ti OR 'leukoencephalopathy, subcortical':ab,ti OR 'leukoencephalopathies, subcortical':ab,ti OR 'subcortical leukoencephalopathies':ab,ti OR 'encephalopathy, subcortical arteriosclerotic':ab,ti OR 'binswangers disease':ab,ti OR 'disease, binswangers':ab,ti OR 'encephalopathy, subcortical, chronic progressive':ab,ti OR 'subcortical encephalopathy, chronic progressive':ab,ti OR 'subcortical leukoencephalopathy':ab,ti OR 'subcortical arteriosclerotic encephalopathy':ab,ti OR 'arteriosclerotic encephalopathy, subcortical':ab,ti OR 'arteriosclerotic encephalopathies, subcortical':ab,ti OR 'encephalopathies, subcortical arteriosclerotic':ab,ti OR 'subcortical arteriosclerotic encephalopathies':ab,ti OR 'binswangers encephalopathy':ab,ti OR 'encephalopathy, binswangers':ab,ti OR 'encephalopathy, binswanger':ab,ti OR 'encephalopathy, chronic progressive subcortical':ab,ti |
| #5 | dementia, AND 'multi infarct' |
| #6 | 'dementia, multi infarct':ab,ti OR 'dementias, multi-infarct':ab,ti OR 'multi-infarct dementias':ab,ti OR 'dementia multi-infarct':ab,ti OR 'dementia multi infarct':ab,ti OR 'dementia multi-infarcts':ab,ti OR 'multi-infarct, dementia':ab,ti OR 'multi-infarcts, dementia':ab,ti OR 'dementia, multiinfarct':ab,ti OR 'dementias, multiinfarct':ab,ti OR 'multiinfarct dementia':ab,ti OR 'multiinfarct dementias':ab,ti OR 'multi-infarct dementia':ab,ti OR 'multi infarct dementia':ab,ti OR 'lacunar dementia':ab,ti OR 'dementia, lacunar':ab,ti OR 'dementias, lacunar':ab,ti OR 'lacunar dementias':ab,ti |
| #7 | 'psci':ab,ti OR 'psd':ab,ti OR 'pscind':ab,ti OR 'post stroke':ab,ti OR 'cognitive impairment cognitive impairment after stroke':ab,ti OR 'post stroke cognitive impairment no dementia':ab,ti OR 'post stroke dementi':ab,ti |
| #8 | #1 OR #2 OR #3 OR #4 OR #5 OR #6 OR #7 |
| #9 | hyperglycemia |
| #10 | 'hyperglycemias':ab,ti OR 'hyperglycemia, postprandial':ab,ti OR 'hyperglycemias, postprandial':ab,ti OR 'postprandial hyperglycemias':ab,ti OR 'postprandial hyperglycemia':ab,ti |
| #11 | 'blood AND glucose |
| #12 | 'blood sugar':ab,ti OR 'sugar, blood':ab,ti OR 'glucose, blood':ab,ti |
| #13 | hypoglycemic AND agents |
| #14 | 'agents, hypoglycemic':ab,ti OR 'hypoglycemic agent':ab,ti OR 'agent, hypoglycemic':ab,ti OR 'antihyperglycemic agent':ab,ti OR 'agent, antihyperglycemic':ab,ti OR 'antihyperglycemics':ab,ti OR 'hypoglycemic':ab,ti OR 'hypoglycemic drugs':ab,ti OR 'drugs, hypoglycemic':ab,ti OR 'hypoglycemics':ab,ti OR 'antihyperglycemic agents':ab,ti OR 'agents, antihyperglycemic':ab,ti OR 'antihyperglycemic':ab,ti OR 'hypoglycemic drug':ab,ti OR 'drug, hypoglycemic':ab,ti OR 'antidiabetics':ab,ti OR 'antidiabetic drug':ab,ti OR 'drug, antidiabetic':ab,ti OR 'antidiabetic drugs':ab,ti OR 'drugs, antidiabetic':ab,ti OR 'antidiabetic agents':ab,ti OR 'agents, antidiabetic':ab,ti OR 'antidiabetic agent':ab,ti OR 'agent, antidiabetic':ab,ti OR 'antidiabetic':ab,ti OR 'hypoglycemic effect':ab,ti OR 'effect, hypoglycemic':ab,ti OR 'hypoglycemic effects':ab,ti OR 'effects, hypoglycemic':ab,ti |
| #15 | #9 OR #10 OR #11 OR #12 OR #13 OR #14 |
| #16 | 'randomized controlled trial':ab,ti OR 'randomized':ab,ti OR 'placebo':ab,ti |
| #17 | #8 AND #15 AND #16 |
